# Phenolic Metabolism Explains Bitterness and Pungency of Extra Virgin Olive Oils

**DOI:** 10.3390/foods14091620

**Published:** 2025-05-03

**Authors:** Sonia Tomé-Rodríguez, Francisco Barba-Palomeque, Mónica Calderón-Santiago, José María Penco-Valenzuela, Feliciano Priego-Capote

**Affiliations:** 1 Department of Analytical Chemistry, University of Córdoba, 14071 Córdoba, Spain; z72toros@uco.es (S.T.-R.); b42casam@uco.es (M.C.-S.); 2 Chemical Institute for Energy and Environment (IQUEMA), Campus of Rabanales, University of Córdoba, 14014 Córdoba, Spain; 3 Maimónides Institute for Biomedical Research (IMIBIC), Reina Sofía University Hospital, 14004 Córdoba, Spain; 4 Consortium for Biomedical Research in Frailty & Healthy Ageing, CIBERFES, Carlos III Health Institute, 28029 Madrid, Spain; 5 Spanish Association of Olive Producing Municipalities (AEMO), Campus Alameda del Obispo, 14004 Córdoba, Spain

**Keywords:** extra virgin olive oil, pungency, bitterness, phenols, secoiridoids, panel test

## Abstract

Organoleptic features allow extra virgin olive oil (EVOO) to be distinguished from other commercial categories and to determine consumer preferences. In this study, we evaluated the influence of the phenolic content on the intensity of two characteristic attributes, namely, bitterness and pungency. The organoleptic analysis was carried out by a panel of trained tasters, who categorized a set of 200 EVOO samples produced in two consecutive crop seasons into three intensity levels (“Delicate”, “Medium”, and “Robust”) according to current regulations. The total phenolic content was correlated with the intensity of both attributes, but a different contribution was identified for individual phenols. For bitterness, aglycone isomers of oleuropein and ligstroside provided over 70% discrimination power (estimated by receiver operating characteristic analysis), while oleocanthal and oleacein were associated with a decrease in bitterness intensity. In addition, the intensity of pungency intensity was related to the content of oleocanthal, oleomissional, and oleokoronal, as they allowed the classification of about 75% of the “Robust” pungency EVOOs. With these premises, it is possible to obtain olive oils with the desired intensity of bitterness and pungency by controlling the factors that influence phenolic metabolism.

## 1. Introduction

Over the years extra virgin olive oil (EVOO) has gained great gastronomic popularity due to its organoleptic characteristics and health benefits [1]. It is worth highlighting the role of the minor fraction and, particularly, the phenolic compounds for their nutraceutical properties [2]. Phenols are responsible for the unique health claim included in the European Regulation EU 432/2012, which is exclusive to olive oil. This claim refers to “olive oil polyphenols contribute to the protection of blood lipids against oxidative stress” and could only be applied if the olive oils contain at least 5 mg of hydroxytyrosol, tyrosol, and their derivatives for a recommended daily intake of 20 g of olive oil [3].

In terms of organoleptic evaluation, fruitiness, pungency, and bitterness are the three main positive attributes evaluated by the panel test. Recently, the International Olive Council (IOC) and the European Commission have classified these attributes according to the intensity of their perception in: “Robust” (median intensity score of all panel tasters > 6.0 on a scale 0–10), “Medium” (median intensity score of all panel tasters between 3.0 and 6.0), and “Delicate” (median intensity score of all panel tasters < 3.0). Complementary, fruitiness is classified as “green fruitiness” and “ripe fruitiness” based on a set of olfactory and gustatory sensory characteristics derived from green and fresh olives or sound and fresh olives, respectively [4,5].

Some studies have previously reported a quantitative association between these attributes and diverse chemical families of compounds in olive oil [6,7]. On the one hand, the fruitiness of EVOO has been explained by the presence of a large number of volatile compounds [8,9,10]. Recently, Tomé-Rodríguez et al. linked the intensity of fruitiness to the main metabolic ratios responsible for the formation of C_5_ and C_6_ volatiles through the lipoxygenase (LOX) pathway. Specifically, the conversion of 3-hexenal to 2-hexenol or 3-hexen-1-ol discriminated between ripe fruitiness (towards the formation of 2-hexenol) and intense fruitiness (towards the formation of 3-hexen-1-ol) [11]. Similarly, Ríos-Reina et al. reported specific volatile markers of fruitiness type using three analytical gas chromatographic methods (HS-SPME–GC–FID, HS-SPME–GC–MS, and TDU–GC–MS) and two data processing strategies (conventional integration and PARADISe software) [7]. Other authors have developed tools to classify the fruitiness intensity of EVOOs (ripe fruitiness or light, medium, and intense green fruitiness) using a high-sensitivity laboratory electronic nose [12].

On the other hand, pungency and bitterness are two sensory attributes that have traditionally been related to the total phenolic content [13,14,15,16], but other studies have linked these attributes to specific secoiridoid derivatives. Oleacanthal has been proposed to be responsible for the pungent sensation in olive oils [17,18,19], while the bitter sensory notes have been linked to the presence of oleuropein and ligstroside derivatives. Demopoulos et al. reported a positive correlation between oleocanthal and oleacein content and pungency and bitterness intensity in Koroneiki virgin olive oils [20] and Gutierrez-Rosales et al. obtained strong correlations between bitterness intensity and oleuropein aglycone, oleacein, and oleocanthal content [21], while the other authors related bitterness to the presence of oleuropein aglycone [22,23].

In recent years, there has been a great interest in developing analytical techniques to measure bitter and pungent attributes in olive oils to support the panel test. However, few studies have related the panel test evaluation of pungency and bitterness to the phenolic profiles of EVOOs. The identification of the phenols responsible for both organoleptic attributes would allow the selection of EVOOs with desired levels of pungency and bitterness. With these premises, the aims of this study were: (i) to find patterns of association between the intensity of these two gustatory attributes, determined by a trained taster panel, and the phenolic profile established by individual determination using LC–MS/MS; and (ii) to explain the intensity of bitterness and pungency by changes occurring in the metabolism of secoiridoids. In order to increase the representativeness of this study, a set of 200 high quality EVOOs produced in two consecutive harvest seasons was selected.

## 2. Materials and Methods

### 2.1. Samples and Sensory Analysis

A set of 200 EVOO samples produced in two consecutive agronomic seasons (2021/2022 and 2022/2023) of different cultivars (monovarietal and blended) and geographical origin were used in this study. The samples were provided by the organizing committee of the EVOOLEUM World’s Top 100 EVOO Guide (Mercacei and AEMO, Spain), corresponding to the top 100 EVOOs included in the 2022 and 2023 Edition Guides. According to the rules of the competition, all the samples submitted had to be certified as extra virgin by an accredited laboratory, guaranteeing the absence of any organoleptic defects. It is worth mentioning that top 100 EVOOs of each edition were selected by the panel of tasters from a total of 450 EVOOs. This selection was based on organoleptic analysis, which means that the quality of top 100 EVOOs was exceptional. Information on the samples is shown in Table S1.

The organizing committee of the EVOOLEUM competition coordinated the organoleptic evaluation of the EVOO samples, which was carried out by a group of twenty-five professional tasters. Each EVOO sample (100 samples × 2 years) was evaluated by five tasters in accordance with the recommendations of the IOC method [5]. Thus, each taster evaluated 20 randomly coded EVOOs in five sessions (4 samples per session) organized on two consecutive days (3 + 2). The EVOO samples were evaluated quantitatively in terms of bitterness and pungency. For this purpose, each taster gave an intensity score for each attribute on a scale of 0–10. For the development of this research, the organizing committee gave the median of all the individual scores provided by the panel testers. The EVOOs were categorized according to the intensity of these two attributes as “Robust”, “Medium”, and “Delicate” according to the median of all the individual scores provided by the panel tasters.

### 2.2. Reagents and Standards

Mass spectrometry (MS)-grade methanol (MeOH) from Fisher Scientific (Hampton, NH, USA) and *n*-hexane from Scharlab (Barcelona, Spain) were used for the determination and quantification of the phenolic compounds in the samples. MS grade formic acid, also from Fisher Scientific was used as ionization agent. Deionized water (18 MΩ·cm) from a Millipore Milli-Q water purification system (Bedford, MA, USA) was used to prepare both mobile phases and the hydroalcoholic mixture used as extractant.

Hydroxytyrosol and tyrosol were purchased in Extrasynthese (Genay, France), secoiridoid derivatives oleacein (3,4-DHPEA-EDA) and oleocanthal (p-HPEA-EDA) were acquired from Phytolab (Vestenbergsgreuth, Germany) and oleuropein and ligstroside aglycones from TRC (Ontario, Canada, Greece). Naringenin from Sigma-Aldrich (St. Louis, MO, USA) was used as internal standard (IS) to control the LC–MS/MS performance during the analysis of all samples.

### 2.3. Determination of Phenolic Compounds in EVOO

Phenolic compounds were isolated by liquid-liquid extraction according to a previously published protocol [24]. A 0.5 g aliquot of oil was vortexed with 250 µL of *n*-hexane for 30 s. Then, 2 mL of 80:20 (*v*/*v*) MeOH:water with the IS (1 µg/mL) was added and shaken for 2 min and the hydroalcoholic phase was separated by centrifugation. Phenolic extraction was performed quantitatively under these conditions as previously reported [25,26]. The phenolic extract was diluted 1:25 (*v*/*v*) before injecting 5 µL into a Thermo Scientific UltiMate 3000 series LC system coupled to a Thermo Scientific QqQ TSQ Quantum™ Access MAX detector (Waltham, MA, USA). Three replicates per sample were analyzed.

MS detection was performed using MS/MS in multiple reaction monitoring (MRM) mode for selective transitions from the precursor ion to the representative product ions for each analyte. The MRM parameters for the determination of the target phenols are given in Table S2.

### 2.4. Quantitative Determination of Phenolic Compounds and Statistical Analysis

Calibration curves were established using refined sunflower oil fortified with different concentrations of the phenolic standards (five concentrations from 1 mg/kg to 20 mg/kg). Each concentration level, prepared in triplicate, was analyzed after application of the complete procedure including sample preparation. The calibration equations were used to calculate the absolute concentration of the target phenols in the EVOO samples (Table S3).

Statistical analysis was performed with R (version 4.3.1., http://www.r-project.org/, accessed on 16 March 2024). Specifically, Kruskal–Wallis and pairwise Wilcoxon tests with a Bonferroni correction (*p*-value < 0.05) were used to detect significant differences between categories of organoleptic characteristics and to evaluate differences in phenolic content between the two harvesting seasons. The Spearman correlation was also used to find associations between the concentration of phenolics and the intensity values of the two sensory characteristics. Partial Least Square Discriminant Analysis (PLS-DA) was used to study the discrimination of EVOOs based on the three categories defined for each attribute. The area under the curve (AUC) of multiclass receiver operating characteristic (ROC) curves was calculated using the pROC R package (version 1.18.5) [27].

## 3. Results and Discussion

### 3.1. Variability in Phenolic Composition and Organoleptic Attributes

As mentioned above, the bitterness and pungency of EVOOs have often been explained by the total phenolic content [13,28]. However, few studies have focused on the association of individual phenols with these two attributes. In this study, EVOOs were classified according to the intensity of bitterness and pungency as determined by the tasting panel. The classification protocol is described in the European Commission Regulation 2022/2104 and considers the following three groups: “Robust” (median intensity score of all panel tasters > 6.0 on a scale 0–10), “Medium” (median intensity score of all panel tasters between 3.0 and 6.0), and “Delicate” (median intensity score of all panel tasters < 3.0). The three categories were well represented in terms of number of samples for the bitterness attribute, but only 11 EVOOs were classified as “Delicate” in pungency. This low representativeness of the “Delicate” pungency group is a limitation of this study, explained by the selection of the tasters, since pungency is a characteristic of high quality EVOOs. Statistical analysis revealed significant differences in the three bitterness groups by comparing the two seasons (Table 1). Thus, EVOOs produced in the 2022–2023 crop season were characterized by a more intense bitterness than those produced in the previous season (*p*-value < 0.001), while significant differences were found only in the “Robust” category for pungency (*p*-value < 0.001).

Despite these differences in the two consecutive seasons, no variation was observed in the total phenolic content of EVOOs (Table 2, Figure S1) with a mean value of 437 mg/kg and a similar distribution in quartiles. Considering the individual phenolic contents, hydroxytyrosol, oleuropein aglycone, and oleacein were significantly higher in the first harvesting season, while oleomissional was significantly higher (*p*-value < 0.001) in the second season. No significant differences were found for tyrosol, ligstroside aglycone, oleokoronal and oleocanthal.

In addition, a correlation analysis using Spearman’s test was carried out to find out possible relationships between organoleptic attributes and phenolic concentration (Table S4). Bitterness intensity showed a positive correlation with total phenolic content and oleuropein aglycone (R = 0.46 and R = 0.38, respectively; *p*-value < 0.001) followed by ligstroside aglycone, oleomissional, and oleokoronal (R = 0.35). These results agree with those reported by Cui et al., who pointed out that no bitter taster receptors are activated by oleacein and oleocanthal [29]. On the other hand, total phenolic content followed by ligstroside aglycone and oleokoronal were positively correlated with pungency (R = 0.39 and R = 0.30, respectively; *p*-value < 0.001). Despite these high levels of significance, the correlation coefficients were less than 0.5. Based on these premises, the contribution of phenolic to bitterness and pungency seems to be explained by several compounds. Bitterness and pungency also showed a positive correlation (R = 0.69; *p*-value < 0.001), which means that both attributes are strongly related.

### 3.2. Influence of the EVOO Phenolic Profile on the Intensity of Bitterness and Pungency

**Figure 1:**
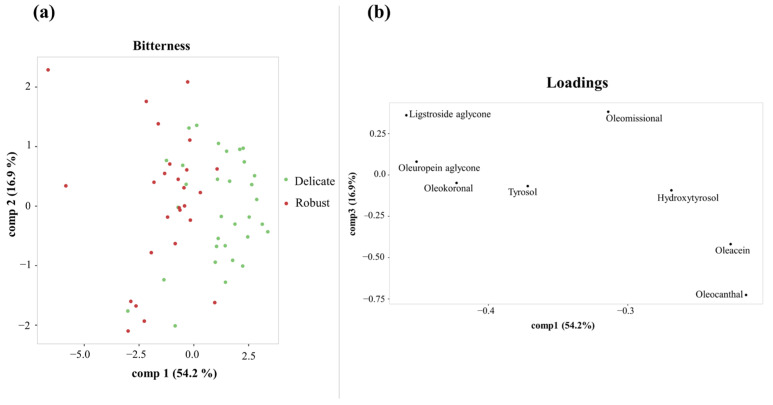
PLS-DA scores plot (**a**) and loadings plot (**b**) for discriminating “Robust” and “Delicate” bitterness groups using the first and third components.

After evaluating the variability in organoleptic characteristics and phenolic content, a multivariate supervised analysis by PLS-DA was performed to evaluate the ability of the phenolic profile to discriminate the three bitterness categories assigned by the European Commission regulation (Figure S2). The EVOOs with “Medium” intensity described a large dispersion in the PLS plot, but the EVOOs with “Delicate” and “Robust” bitterness were well discriminated. For this reason, we performed a second PLS-DA analysis restricted to EVOOs belonging to the “Delicate” (*n* = 32) and “Robust” (*n* = 26) bitterness categories. The PLS-DA scores plot (Figure 1a) showed a clear distinction between the two groups along the first component. Also, the loadings plot (Figure 1b) showed the discriminatory role of the closed monoaldehyde form of both aglycones (oleuropein and ligstroside aglycones) and the open aldehyde form of the ligstroside aglycone (oleokoronal) for EVOOs categorized with “Robust” bitterness, whereas oleacein and oleocanthal were distributed with the “Delicate” bitterness group. Regarding the pungency attribute, PLD-DA did not reveal any clear discrimination between the three groups or between the “Medium” and “Robust” intensity groups (Figure S3).

**Figure 2:**
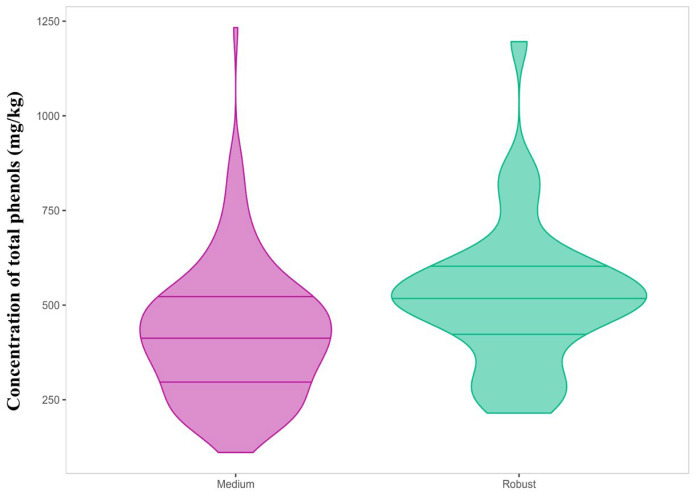
Violin plot of the total phenolic content (mg/kg) for “Medium” and “Robust” pungency categories.

However, Kruskal–Wallis and pairwise Wilcoxon tests (adjusted Bonferroni *p* < 0.05) were used as complementary analysis to find differences between the groups. They revealed significant differences in the total phenolic content for the two groups correctly represented by pungency (Table 3, Figure 2).

**Figure 3:**
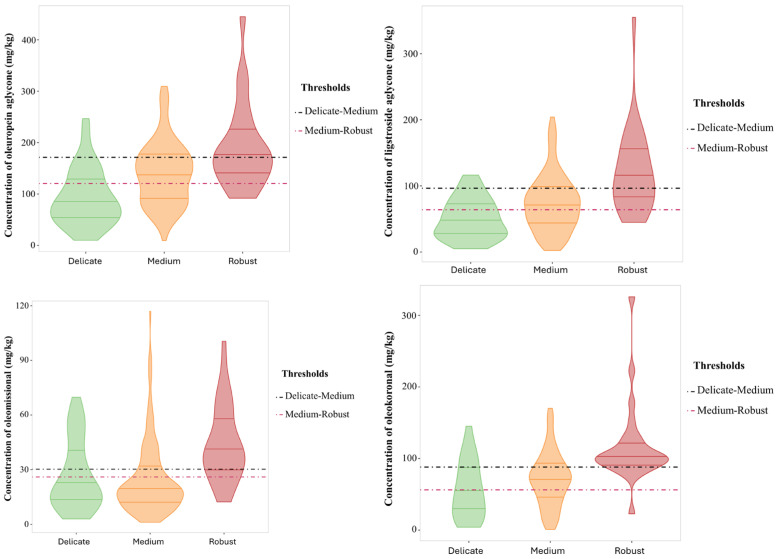
Violin plot for the concentration of secoiridoids (mg/kg) with significant differences between the bitterness groups. Threshold concentrations are included to discriminate between “Delicate” and “Medium” (pink line) and between “Medium” and “Robust” (black line).

The “Robust” pungency group had a total phenolic content of 522 ± 189 mg/kg as compared to the “Medium” group with 422 ± 175 mg/kg. Significant differences were also reported for three individual phenols, namely, the open isomers, oleomissional and oleokoronal (*p*-values 0.0015 and 0.05, respectively), and oleocanthal (*p*-value = 0.021). These three compounds were more concentrated in the “Robust” pungency group, despite other studies associating this organoleptic property to the presence of oleocanthal [17,18]. Similarly, the three groups established for bitterness showed significant differences in the total phenolic content, but also in the individual content of specific phenols such as the aglycone isomers (Table 3, Figure 3). Thus, the total phenolic content increased linearly with the intensity of bitterness, with the mean content in the “Robust” group being 621 ± 223 mg/kg while the “Delicate” group reported 327 ± 144 mg/kg. Among the individual phenols, only oleuropein aglycone and ligstroside aglycone showed significant variations with bitterness intensity with clear differences between the three categories. The content in oleuropein aglycone in the “Delicate” group was 93.3 ± 55.4 mg/kg as compared to 190 ± 82 mg/kg in the “Robust” group. Similarly, ligstroside aglycone was found to be 50.8 ± 30.3 mg/kg in the “Delicate” group as compared to 121 ± 64 mg/kg in the “Robust” group. These results agree with those obtained by Cui et al., who found a high stimulation ability of bitter receptors for these two phenols using a calcium mobilization functional assay [29]. Hydroxytyrosol showed a similar behavior to both aglycones but the range of variation in concentration was reduced. Oleomissional, oleokoronal, and oleocanthal also showed statistical differences, but only allowed differentiation of the “Robust” bitterness group.

### 3.3. Capability of Phenols to Discriminate Bitterness and Pungency Intensity of EVOOs

A complementary analysis of the ability of phenols to discriminate the intensity of bitterness and pungency in EVOOs was performed using receiver operating characteristic (ROC) curves and pairwise comparisons [27]. This evaluation was multiclass for bitterness due to the categorization into three intensity groups, while for pungency only two groups were considered due to the reduced representativeness of the “Delicate” group. For bitterness, ligstroside aglycone, oleuropein aglycone, oleokoronal, and oleomissional gave multiclass AUC percentages above 70%, while oleacein and oleocanthal gave values below 60% (Table 4). These results were complemented by the pairwise comparison reporting AUC values for discriminating between two groups, namely, “Delicate” vs. “Medium”; “Medium” vs. “Robust”; and “Delicate” vs. “Robust”. Comparison of the two extreme groups, “Delicate” vs. “Robust”, clearly showed the discrimination ability of the aglycone isomers of oleuropein and ligstroside with AUC values ranging from 75.6% to 88.3%. The highest AUC percentages were found for oleuropein and ligstroside aglycones (closed forms) with 86.2 and 88.3, respectively. It is worth mentioning that hydroxytyrosol and tyrosol, simple phenols, also gave AUC values above 80, but their concentration variability ranges were particularly limited. On the other hand, the AUC values for oleacein and oleocanthal for discriminating the two extreme groups were the lowest among those monitored for individual phenols. A similar pattern was observed when comparing “Delicate” and “Medium” groups, as the highest AUC values were found for oleuropein aglycone (72.7%) and ligstroside aglycone (66.2%), followed by hydroxytyrosol (67.2%). The comparison between the “Medium” and “Robust” groups allowed the detection of the open aglycone isomers, oleomissional (79.8%) and oleokoronal (80.4%), as the phenols with the highest AUC values followed by the two closed isomers, oleuropein aglycone (68.7%) and ligstroside aglycone (74.0%). The simple phenols also showed high AUC values, 67.9% and 70.5% for hydroxytyrosol and tyrosol, respectively, whereas the lowest AUC values were obtained for oleacein and oleocanthal.

For pungency, the discrimination AUC values were lower than those found for bitterness. Thus, the total phenolic content gave an AUC of 67.0% when comparing the “Medium” and “Robust” pungency groups, while the three individual phenols that gave significant differences were the top 3 phenols with the highest discriminatory ability. These were oleomissional (68.0%), oleokoronal (62.0%), and oleocanthal (64.0%).

### 3.4. Association of Bitterness and Pungency with Phenolic Content

**Figure 4:**
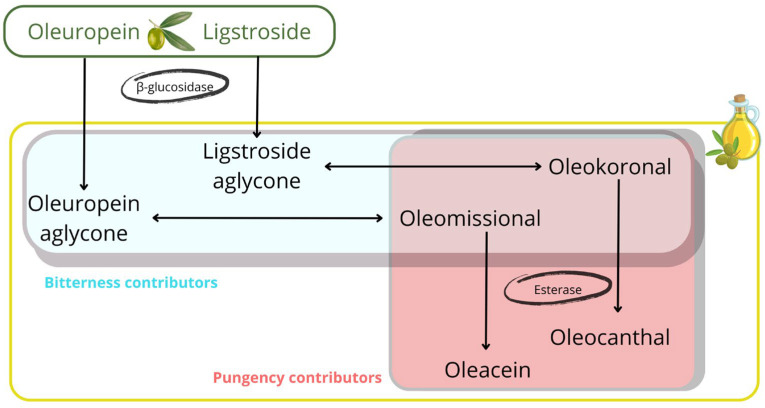
Pathway for the synthesis of the main secoiridoids found in olive oil with indication of the main contributors of bitterness and pungency.

Considering the synthetic pathway involved in the formation of the main secoiridoid derivatives found in olive oil (Figure 4), the compounds with the highest ability to explain bitterness are the aglycone isomers of oleuropein and ligstroside, which are released by enzymatic conversion of the oleuropein and ligstroside substrates by β-glucosidase. The closed isomers are in chemical equilibrium with the open forms, the latter being the substrates for conversion by the esterase enzyme to oleacein and oleocanthal [30]. According to the results reported in this study, the predominance of these last two secoiridoids in the phenolic profile is associated with a decrease in bitterness intensity. Regarding the role of the aglycone isomers, the opening of the closed cycle in the aglycones also seems to play a relevant role in the bitterness intensity. Although the concentration of both isomeric forms of oleuropein and ligstroside are associated with bitterness intensity, the closed forms provided a strong concentration effect on the intensity of this attribute, in agreement with the study reported by Cui et al., who attributed the highest ability to activate bitter taste receptors to the closed isomers [30]. On the other hand, oleomissional had the ability to activate only one of the bitter taste receptors (TAS2R8) whereas oleocanthal and oleacein did not activate any of these receptors (TAS2Rs). These results were consistent with those obtained in our study, supported by the violin plots and the threshold concentrations for discriminating the three bitter intensity groups (Figure 3). Thus, the thresholds of oleuropein and ligstroside aglycones (closed forms) for discriminating the “Delicate” and “Medium” groups allowed the classification of almost 75% of the EVOOs with “Delicate” bitterness against about 60% of the EVOOs with “Medium” bitterness. On the other hand, the thresholds of the two closed aglycones allowed the classification of about 75% of the “Medium” bitterness EVOOs and less than 50% of the “Robust” bitterness EVOOs.

The discriminatory power capability was less significant for the open isomers, as they mainly classified “Robust” bitterness EVOOs. Thus, the thresholds for oleomissional and oleokoronal allowed us to discriminate about 75% of the “Robust” bitterness EVOOs. These results indicate that the closed isomers of oleuropein and ligstroside aglycones are the strongest contributors to EVOO bitterness. However, the open forms, oleomissional and oleokoronal, also contribute to “Robust” bitterness EVOOs, probably because they are positively correlated with the presence of the closed isomers. This finding supports the conclusions reported by Servili et al. concerning the relationship between the attributes and ring opening in aglycone secoiridoids [31].

For pungency, we found a similar effect to that observed for bitterness (Figure S4). Thus, the threshold concentrations determined for oleomissional and oleokoronal allowed the classification of about 75% of the “Medium” pungency EVOOs and more than 50% of the “Robust” pungency EVOOs. Complementarily, oleocanthal, formed by the esterase conversion of oleokoronal, reported a discrimination threshold that correctly classified about 50% of “Medium” pungency EVOOs and almost 75% of “Robust” pungency EVOOs. Therefore, it seems that oleomissional and oleokoronal are mainly responsible for the pungency of EVOOs, but oleocanthal also contributes to the discrimination of “Robust” pungency EVOOs, as reported by Peyrot des Ganchons et al. [19] and Andrewes et al. [17], probably because this phenol is positively correlated with the presence of oleokoronal. Overall, the first part of this synthetic pathway regulates bitterness, while the second part is responsible for the pungency attribute. This explanation supports the high level of correlation reported between the intensity of the two attributes.

## 4. Conclusions

Phenolic composition was associated with the intensity of bitterness and pungency in EVOOs. Oleuropein and ligstroside aglycone were positively correlated with the intensity of bitterness, although oleomissional and oleokoronal also contributed to discriminate the “Robust” intensity group. On the other hand, oleacein and oleocanthal were associated with the lowest intensity of bitterness. Complementary, oleomissional, oleokoronal, and oleocanthal showed the highest ability to discriminate between the “Medium” and “Robust” pungency groups. The metabolic pathway involved in the synthesis of the main secoiridoids can therefore explain the intensity of these two attributes, which are of particular relevance in the organoleptic evaluation of EVOOs. With these premises, the control of the factors that influence the metabolism of secoiridoids would make it possible to obtain olive oils with the desired intensity of bitterness and pungency, which is of interest to the olive oil industry in order to prepare products according to consumer preferences.

## Figures and Tables

**Table 1:** Organoleptic evaluation of bitterness and pungency in EVOOs selected in the two harvesting seasons. Letters indicate significant differences between the three groups for bitterness and pungency (*p* < 0.05). Asterisks indicate significant differences between the groups.

Crop Season	Bitterness	Pungency
2021–2022		
Delicate (n_bitterness_ = 7; n_pungency_ = 3)	2.57 ± 0.18 ^c (***)^	2.97 ± 0.06 ^c^
Medium (n_bitterness_ = 86; n_pungency_ = 82)	4.42 ± 0.64 ^b (***)^	4.82 ± 0.65 ^b^
Robust (n_bitterness_ = 5; n_pungency_ = 13)	6.32 ± 0.14 ^a (***)^	6.49 ± 0.34 ^a (***)^
2022–2023		
Delicate (n_bitterness_ = 25; n_pungency_ = 8)	2.86 ± 0.33 ^c^	2.69 ± 0.53 ^c^
Medium (n_bitterness_ = 54; n_pungency_ = 64)	4.99 ± 0.83 ^b^	4.96 ± 0.85 ^b^
Robust (n_bitterness_ = 21; n_pungency_ = 27)	6.86 ± 0.36 ^a^	6.98 ± 0.35 ^a^

**Table 2:** Mean phenolic content (expressed in mg/kg) and standard deviation determined in EVOO samples of each crop season.

Crop Season	Hydroxytyrosol (*)	Tyrosol	Oleuropein Aglycone (***)	Ligstroside Aglycone	Oleomissional (***)	Oleokoronal	Oleacein (**)	Oleocanthal	Total Phenols
2021–2022	5.67 ± 2.81	8.16 ± 3.47	151 ± 64	73.1 ± 40.5	17.8 ± 12.7	69.8 ± 36.0	55.5 ± 41.9	56.3 ± 44.9	437 ± 159
2022–2023	5.17 ± 4.15	8.34 ± 3.91	127 ± 70	79.8 ± 55.2	36.9 ± 22.6	79.5 ± 50.4	45.8 ± 46.7	54.4 ± 38.6	437 ± 200

* Content of phenol with significant differences between crop seasons (*p* < 0.05); ** Content of phenol with significant differences between crop seasons (*p* < 0.01); *** Content of phenol with significant differences between crop seasons (*p* < 0.001).

**Table 3:** Mean concentration of phenols (expressed in mg/kg) in the total set of samples in each category of bitterness and pungency. Lowercase letters indicate significant differences (*p* < 0.05) between intensity groups for each attribute.

	Hydroxytyrosol	Tyrosol	Oleuropein Aglycone	Ligstroside Aglycone	Oleomissional	Oleokoronal	Oleacein	Oleocanthal	Total Phenols
Bitterness									
Delicate	4.16 ± 4.72 ^c^	6.80 ± 3.63 ^b^	93.3 ± 55.4 ^c^	50.8 ± 30.3 ^c^	26.8 ± 19.0 ^b^	56.0 ± 39.4 ^b^	37.8 ± 29.7	51.5 ± 33.5 ^ab^	327 ± 144 ^c^
Medium	5.41 ± 3.31 ^b^	8.18 ± 3.65 ^b^	140 ± 61 ^b^	74.1 ± 43.1 ^b^	24.4 ± 19.6 ^b^	71.2 ± 37.7 ^b^	46.4 ± 33.5	52.4 ± 40.8 ^b^	428 ± 153 ^b^
Robust	7.00 ± 2.50 ^a^	10.4 ± 3.0 ^a^	190 ± 82 ^a^	121 ± 64 ^a^	44.2 ± 21.1 ^a^	117 ± 56 ^a^	68.6 ± 59.9	75.6 ± 50.9 ^a^	621 ± 223 ^a^
Pungency									
Delicate	5.76 ± 7.44	7.57 ± 4.60	101 ± 70 ^b^	54.2 ± 32.4	25.8 ± 19.37 ^ab^	56.3 ± 41.7 ^b^	38.6 ± 15.8	56.6 ± 32.6 ^ab^	346 ± 137 ^b^
Medium	5.26 ± 3.33	8.25 ± 3.75	137 ± 66 ^ab^	75.1 ± 48.8	25.0 ± 19.94 ^b^	72.5 ± 43.4 ^b^	47.6 ± 40.1	51.0 ± 39.8 ^b^	422 ± 175 ^b^
Robust	5.84 ± 2.81	8.49 ± 3.28	158 ± 74 ^a^	89.2 ± 48.1	35.5 ± 20.52 ^a^	88.8 ± 44.8 ^a^	64.7 ± 61.0	71.8 ± 47.5 ^a^	522 ± 189 ^a^

**Table 4:** AUC values (%) estimated for phenolic compounds to discriminate intensity groups of bitterness (multiclass AUC) and pungency (“Medium” versus “Robust”). AUC values (%) and threshold concentrations (mg/kg) obtained by pairwise comparison of bitterness intensity groups are also provided.

Compound	Bitterness						Pungency
	Multiclass AUC	AUC “Delicate” vs. “Medium”	AUC “Medium” vs. “Robust”	AUC “Delicate” vs. “Robust”	Threshold “Delicate” vs. “Medium”	Threshold “Delicate” vs. “Robust”	AUC “Medium” vs. “Robust”
Hydroxytyrosol	71.8	67.2	67.9	80.2	3.78	4.77	56.6
Tyrosol	72.2	62.4	70.5	83.7	7.31	8.48	54.5
Oleuropein aglycone	75.9	72.7	68.7	86.2	120	128	59.3
Oleomissional	69.5	53.1	79.8	75.6	26.0	29.3	67.9
Ligstroside aglycone	76.2	66.2	74.0	88.3	64.0	63.9	58.7
Oleokoronal	75.1	61.5	80.4	83.5	56.2	87.0	62.2
Oleacein	58.8	57.7	55.7	63.0	27.9	37.6	59.1
Oleocanthal	59.9	53.1	65.0	61.7	41.9	47.5	63.9
Total phenols	78.2	68.6	77.6	88.3	362	405	67.3

## Data Availability

The original contributions presented in the study are included in the article/Supplementary Material, further inquiries can be directed to the corresponding author.

## Supplementary Materials

The following supporting information can be downloaded at: https://www.mdpi.com/article/10.3390/foods14091620/s1, Table S1. List of EVOO samples analyzed in this study. Bitterness and pungency are categorized in three groups according to the ranges established by Regulation (EU) 2022/2104 of 29 July 2022. Table S2. LC–MS/MS parameters for determination of phenolic compounds. Table S3. Calibration models used for quantitative analysis of phenols. Table S4. Correlation coefficients between the intensity of bitterness and pungency and the concentration of phenolic compounds. Statistical significance is denoted as follows: *, *p* < 0.05; **, *p* < 0.01; ***, *p* < 0.001. Figure S1. Distribution of the total phenolic content in EVOOs selected in each crop season. Figure S2. PLS-DA scores plot relative to the three bitterness categories. Figure S3. PLS-DA scores plot relative to the three pungency categories (a) and the “Medium” versus “Robust” categories (b). Figure S4. Violin plot for the concentration of secoiridoids (mg/kg) with significant differences between pungency groups. Threshold concentrations are included for discrimination of “Medium” and “Robust”.
