# Supplementary material for: Phenolic Metabolism Explains Bitterness and Pungency of Extra Virgin Olive Oils
Source: Foods. 2025 May 3;14(9):1620. doi: 10.3390/foods14091620 (PMC12071655; doi:10.3390/foods14091620)
Supplement: Supplementary file 1 [file foods-14-01620-s001.zip › foods-3597981-supplementary.pdf]

Table S1. List of EVOO samples analyzed in this study. Bitterness and pungency are categorized in three groups according to the ranges established by Regulation (EU) 2022/2104 of 29 July 2022.

Sample	Cultivar	Geographical origin	Agronomic season	Bitterness intensity	Pungency intensity
**1**	Picual	Spain	2021/2022	Medium	Medium
**2**	Blend	Spain	2021/2022	Medium	Medium
**3**	Picual	Spain	2021/2022	Medium	Medium
**4**	Blend	Brazil	2021/2022	Medium	Medium
**5**	Picual	Spain	2021/2022	Delicate	Medium
**6**	Blend	Spain	2021/2022	Medium	Medium
**7**	Blend	Spain	2021/2022	Medium	Medium
**8**	Blend	France	2021/2022	Medium	Medium
**9**	Picual	Spain	2021/2022	Medium	Medium
**10**	Bosana	Italy	2021/2022	Medium	Medium
**11**	Picual	Spain	2021/2022	Medium	Medium
**12**	Picual	Spain	2021/2022	Delicate	Medium
**13**	Picual	Spain	2021/2022	Medium	Medium
**14**	Blend	Italy	2021/2022	Medium	Medium
**15**	Blend	Italy	2021/2022	Medium	Medium
**16**	Picual	Spain	2021/2022	Medium	Medium
**17**	Picual	Spain	2021/2022	Medium	Medium
**18**	Hojiblanca	Spain	2021/2022	Medium	Medium
**19**	Royal	Spain	2021/2022	Medium	Medium
**20**	Picual	Spain	2021/2022	Medium	Medium
**21**	Coratina	South Africa	2021/2022	Medium	Medium
**22**	Hojiblanca	Spain	2021/2022	Medium	Medium
**23**	Picual	Spain	2021/2022	Medium	Medium
**24**	Carolea	Italy	2021/2022	Medium	Medium
**25**	Koroneiki	Greece	2021/2022	Medium	Medium
**26**	Picual	Spain	2021/2022	Medium	Medium
**27**	Picholine Marocaine	Morocco	2021/2022	Medium	Medium
**28**	Dritta	Italy	2021/2022	Medium	Medium
**29**	Blend	Tunisie	2021/2022	Medium	Medium
**30**	Chetoui	Tunisie	2021/2022	Medium	Medium
**31**	Blend	Spain	2021/2022	Medium	Medium
**32**	Picual	Spain	2021/2022	Medium	Medium
**33**	Picual	Spain	2021/2022	Medium	Medium
**34**	Coratina	Italy	2021/2022	Medium	Medium
**35**	Peranzana	Italy	2021/2022	Medium	Medium
**36**	Blend	Italy	2021/2022	Medium	Medium
**37**	Hojiblanca	Spain	2021/2022	Medium	Medium
**38**	Picual	Spain	2021/2022	Medium	Medium
**39**	Arbequina	Spain	2021/2022	Medium	Medium
**40**	Picual	Spain	2021/2022	Delicate	Delicate
**41**	Blend	Spain	2021/2022	Medium	Medium
**42**	Hojiblanca	Spain	2021/2022	Medium	Robust
**43**	Picual	Spain	2021/2022	Medium	Medium

**44**	Picual	Spain	2021/2022	Medium	Medium
**45**	Blend	Croatia	2021/2022	Medium	Medium
**46**	Picual	Spain	2021/2022	Medium	Medium
**47**	Arbequina	Spain	2021/2022	Medium	Medium
**48**	Blend	Spain	2021/2022	Medium	Medium
**49**	Picuda	Spain	2021/2022	Medium	Medium
**50**	Hojiblanca	Spain	2021/2022	Medium	Medium
**51**	Picual	Spain	2021/2022	Medium	Medium
**52**	Picual	Spain	2021/2022	Medium	Medium
**53**	Blend	Spain	2021/2022	Medium	Medium
**54**	Hojiblanca	Spain	2021/2022	Medium	Medium
**55**	Picual	Spain	2021/2022	Medium	Medium
**56**	Blend	Spain	2021/2022	Medium	Medium
**57**	Picual	Spain	2021/2022	Medium	Medium
**58**	Picual	Spain	2021/2022	Medium	Medium
**59**	Arbequina	Spain	2021/2022	Delicate	Medium
**60**	Blend	Spain	2021/2022	Medium	Medium
**61**	Pico limón	Spain	2021/2022	Medium	Medium
**62**	Blend	Croatia	2021/2022	Medium	Medium
**63**	Picual	Spain	2021/2022	Medium	Medium
**64**	Blend	Spain	2021/2022	Medium	Medium
**65**	Picual	Spain	2021/2022	Medium	Medium
**66**	La Roda de andalucía	Spain	2021/2022	Delicate	Delicate
**67**	Frantoio	Spain	2021/2022	Medium	Medium
**68**	Cornicabra	Spain	2021/2022	Robust	Medium
**69**	Nevadillo negro	Spain	2021/2022	Delicate	Delicate
**70**	Picual	Spain	2021/2022	Medium	Medium
**71**	Ogliarola	Italy	2021/2022	Robust	Robust
**72**	Hojiblanca	Spain	2021/2022	Medium	Robust
**73**	Manzanilla cacereña	Spain	2021/2022	Delicate	Delicate
**74**	Frantoio	Italy	2021/2022	Medium	Medium
**75**	Coratina	Italy	2021/2022	Delicate	Medium
**76**	Blend	Spain	2021/2022	Medium	Medium
**77**	Hojiblanca	Spain	2021/2022	Medium	Medium
**78**	Picual	Spain	2021/2022	Medium	Medium
**79**	Blend	France	2021/2022	Medium	Medium
**80**	Ortice	Italy	2021/2022	Medium	Robust
**81**	Koroneiki	Greece	2021/2022	Medium	Robust
**82**	Bosana	Italy	2021/2022	Robust	Robust
**83**	Blend	Spain	2021/2022	Medium	Medium
**84**	Picual	Portugal	2021/2022	Medium	Robust
**85**	Koroneiki	Greece	2021/2022	Medium	Robust
**86**	Picual	Spain	2021/2022	Medium	Medium
**87**	Koroneiki	Greece	2021/2022	Medium	Medium
**88**	Arbequina	Spain	2021/2022	Medium	Medium
**89**	Blend	Italy	2021/2022	Medium	Medium

**90**	Coratina	Italy	2021/2022	Medium	Medium
**91**	Hojiblanca	Spain	2021/2022	Medium	Medium
**92**	Picual	Spain	2021/2022	Medium	Medium
**93**	Tonda Iblea	Italy	2021/2022	Medium	Robust
**94**	Blend	Spain	2021/2022	Medium	Robust
**95**	Coratina	Italy	2021/2022	Robust	Robust
**96**	Hojiblanca	Spain	2021/2022	Medium	Medium
**97**	Arbosana	Spain	2021/2022	Medium	Medium
**98**	Arbequina	Spain	2021/2022	Medium	Medium
**99**	Picual	Spain	2021/2022	Medium	Robust
**100**	Blend	Croatia	2021/2022	Robust	Robust
**101**	Hojiblanca	Spain	2022/2023	Delicate	Delicate
**102**	Koroneiki	España	2022/2023	Delicate	Delicate
**103**	Ortice	Italy	2022/2023	Delicate	Delicate
**104**	Tríley	Turkey	2022/2023	Medium	Delicate
**105**	Picual	Spain	2022/2023	Delicate	Delicate
**106**	Arbequina	Spain	2022/2023	Delicate	Delicate
**107**	Leccino	China	2022/2023	Delicate	Delicate
**108**	Picual	Spain	2022/2023	Delicate	Delicate
**109**	Blend	Spain	2022/2023	Robust	Medium
**110**	Frantoio	Italy	2022/2023	Medium	Medium
**111**	Koroneiki	Greece	2022/2023	Delicate	Medium
**112**	Blend	Brasil	2022/2023	Delicate	Medium
**113**	Picual	Portugal	2022/2023	Medium	Medium
**114**	Nocellara	Italy	2022/2023	Medium	Medium
**115**	Picual	Spain	2022/2023	Medium	Medium
**116**	Blend	Spain	2022/2023	Medium	Medium
**117**	Picual	Spain	2022/2023	Medium	Medium
**118**	Picual	Spain	2022/2023	Medium	Medium
**119**	Blend	Spain	2022/2023	Medium	Medium
**120**	Blend	Portugal	2022/2023	Medium	Medium
**121**	Hojiblanca	Spain	2022/2023	Medium	Medium
**122**	Blend	Spain	2022/2023	Delicate	Medium
**123**	Coratina	China	2022/2023	Delicate	Medium
**124**	Hojiblanca	Spain	2022/2023	Delicate	Medium
**125**	Hojiblanca	Spain	2022/2023	Delicate	Medium
**126**	Picual	Spain	2022/2023	Delicate	Medium
**127**	Tríley	Turkey	2022/2023	Delicate	Medium
**128**	Blend	Spain	2022/2023	Delicate	Medium
**129**	Picual	Spain	2022/2023	Delicate	Medium
**130**	Picholine	Italy	2022/2023	Delicate	Medium
**131**	Picual	Spain	2022/2023	Delicate	Medium
**132**	Picual	Spain	2022/2023	Delicate	Medium
**133**	Coratina	Italy	2022/2023	Medium	Medium
**134**	Picual	Spain	2022/2023	Medium	Medium
**135**	Blend	Slovenija	2022/2023	Medium	Medium
**136**	Picual	Spain	2022/2023	Medium	Medium
**137**	Blend	Portugal	2022/2023	Medium	Medium

**138**	Coratina	Spain	2022/2023	Medium	Medium
**139**	Picual	Spain	2022/2023	Medium	Medium
**140**	Blend	Spain	2022/2023	Medium	Medium
**141**	Frantoio	Spain	2022/2023	Medium	Medium
**142**	Picual	Spain	2022/2023	Medium	Medium
**143**	Chemleli	Tunisie	2022/2023	Medium	Medium
**144**	Vodnjanska Buza	Hrvatska	2022/2023	Medium	Medium
**145**	Picual	Spain	2022/2023	Delicate	Medium
**146**	Picual	Spain	2022/2023	Delicate	Medium
**147**	Blend	Italy	2022/2023	Delicate	Medium
**148**	Picual	Spain	2022/2023	Robust	Medium
**149**	Picual	Spain	2022/2023	Medium	Medium
**150**	Picual	Spain	2022/2023	Medium	Medium
**151**	Picual	Spain	2022/2023	Medium	Medium
**152**	Manzanilla cacereña	Spain	2022/2023	Medium	Medium
**153**	Picual	Spain	2022/2023	Medium	Medium
**154**	Pajarera	Spain	2022/2023	Delicate	Medium
**155**	Ogliarola	Italy	2022/2023	Robust	Medium
**156**	Blend	Portugal	2022/2023	Robust	Medium
**157**	Picual	Spain	2022/2023	Robust	Medium
**158**	Bianchera	Croatia	2022/2023	Robust	Medium
**159**	Ogliarola garganica	Italy	2022/2023	Robust	Medium
**160**	Picual	Spain	2022/2023	Robust	Medium
**161**	Blend	Italy	2022/2023	Medium	Medium
**162**	Coratina	Italy	2022/2023	Medium	Medium
**163**	Coratina	South Africa	2022/2023	Medium	Medium
**164**	Hojiblanca	Spain	2022/2023	Medium	Medium
**165**	Hojiblanca	Spain	2022/2023	Medium	Medium
**166**	Arbequina	Spain	2022/2023	Medium	Medium
**167**	Blend	Portugal	2022/2023	Medium	Medium
**168**	Hojiblanca	Spain	2022/2023	Medium	Medium
**169**	Blend	Spain	2022/2023	Medium	Medium
**170**	Peranzana	Italy	2022/2023	Medium	Medium
**171**	Blend	Spain	2022/2023	Medium	Medium
**172**	Picual	Spain	2022/2023	Medium	Medium
**173**	Coratina	Italy	2022/2023	Robust	Robust
**174**	Picual	Spain	2022/2023	Medium	Robust
**175**	Picual	Spain	2022/2023	Medium	Robust
**176**	Picual	Spain	2022/2023	Medium	Robust
**177**	Memecik	Turkey	2022/2023	Medium	Robust
**178**	Picual	Spain	2022/2023	Medium	Robust
**179**	Arbequina	Spain	2022/2023	Delicate	Robust
**180**	Nocellara del Belice	Italy	2022/2023	Robust	Robust
**181**	Picual	Spain	2022/2023	Robust	Robust
**182**	Picual	Spain	2022/2023	Robust	Robust
**183**	Picual	Spain	2022/2023	Robust	Robust

**184**	Blend	Spain	2022/2023	Robust	Robust
**185**	Picual	Spain	2022/2023	Robust	Robust
**186**	Coratina	Italy	2022/2023	Robust	Robust
**187**	Blend	Portugal	2022/2023	Robust	Robust
**188**	Arbequina	Spain	2022/2023	Medium	Robust
**189**	Royal	Spain	2022/2023	Medium	Robust
**190**	Picholine Marocaine	Morocco	2022/2023	Medium	Robust
**191**	Royal	Spain	2022/2023	Medium	Robust
**192**	Picual	Portugal	2022/2023	Medium	Robust
**193**	Picual	Spain	2022/2023	Medium	Robust
**194**	Blend	Croatia	2022/2023	Robust	Robust
**195**	Cornicabra	Spain	2022/2023	Robust	Robust
**196**	Picual	Spain	2022/2023	Robust	Robust
**197**	Picual	Spain	2022/2023	Robust	Robust
**198**	Blend	Italy	2022/2023	Medium	Robust
**199**	Hojiblanca	Spain	2022/2023	Medium	Robust
**200**	Koroneiki	Israel	2022/2023	Medium	Medium

Table S2. LC–MS/MS parameters for determination of phenolic compounds.

**Compound**	**Retention time (min)**	**Precursor ion (m/z)**	**Product ion (m/z)**	**Collision energy (eV)**	**Tube lens (V)**
Hydroxytyrosol	1.7	153	123	15	80
Tyrosol	2.2	137	106	20	80
Oleacein	3.8	319	69	40	100
Oleomissional	4.0	377	275	10	80
Oleocanthal	4.5	303	59	10	80
Oleokoronal	4.6	361	291	10	110
Naringenin (IS)	5.0	271	119	30	100
Oleuropein aglycone	5.1	377	275	10	80
Ligstroside aglycone	5.4	361	291	10	110

Table S3. Calibration models used for quantitative analysis of phenols.

Compound	Calibration model	R^2	Calibration range
Hydroxytyrosol	y = 0.3734x + 0.4379	0.9864	1-20 mg/kg
Tyrosol	y = 0.0648x + 0.0076	0.9905	1-20 mg/kg
Oleocanthal	y = 0.1773x + 0.3285	0.9839	1-20 mg/kg
Oleacein	y = 0.0309x + 0.0399	0.9969	1-20 mg/kg
Ligstroside aglycone	y = 1.9011x + 3.6379	0.9716	1-20 mg/kg
Oleuropein aglycone	y = 0.5129x + 0.887	0.9928	1-20 mg/kg

Table S4. Correlation coefficients between the intensity of bitterness and pungency and the concentration of phenolic compounds. Statistical significance is denoted as follows: *, p<0.05; **, p<0.01; ***, p<0.001.

	Bitterness	Pungency	Hydroxytyrosol	Tyrosol	Oleuropein aglycone	Oleomissional	Ligstroside aglycone	Oleokoronal	Oleacein	Oleocanthal	Total phenols
**Bitterness**	-	0.69 (***)	0.26 (***)	0.25 (***)	0.38 (***)	0.35 (***)	0.35 (***)	0.35 (***)	0.17 (*)	0.13	0.46 (***)
**Pungency**	0.69 (***)	-	0.15 (*)	0.19 (**)	0.26 (***)	0.28 (***)	0.30 (***)	0.30 (***)	0.16 (*)	0.17 (*)	0.39 (***)

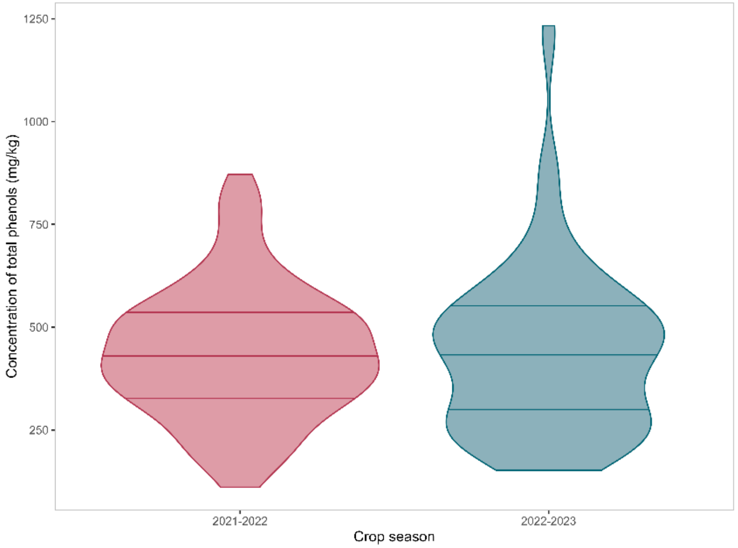

Figure S1. Distribution of the total phenolic content in EVOOs selected in each crop season.

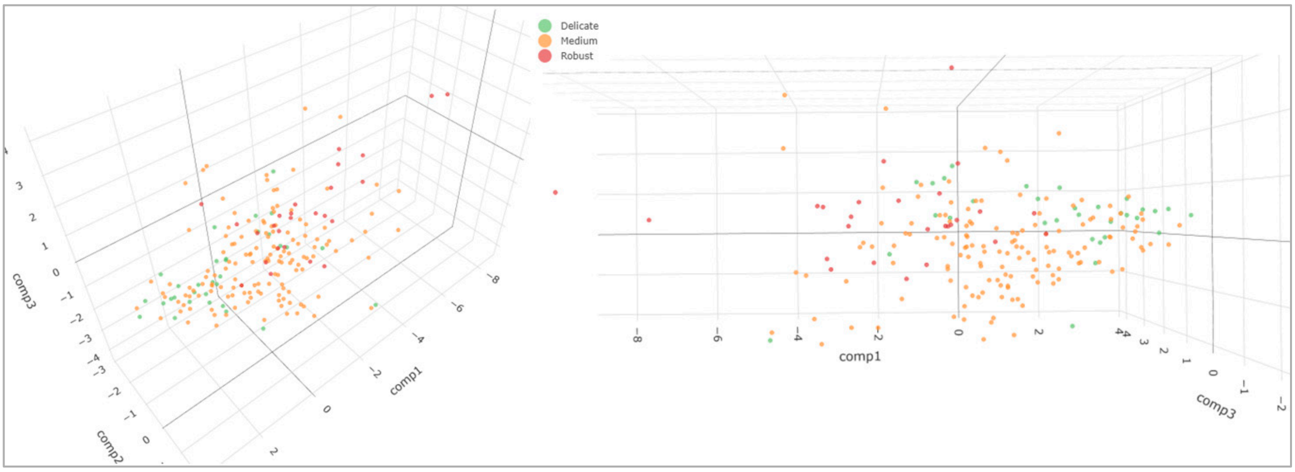

Figure S2. PLS-DA scores plot relative to the three bitterness categories.

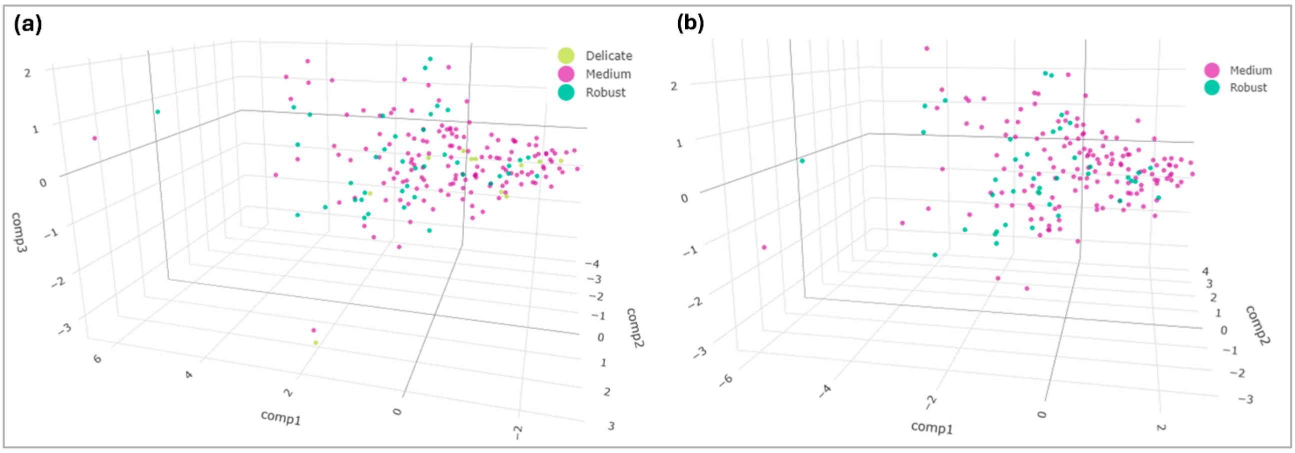

Figure S3. PLS-DA scores plot relative to the three pungency categories (a) and the "*Medium*" versus "*Robust*" categories (b).

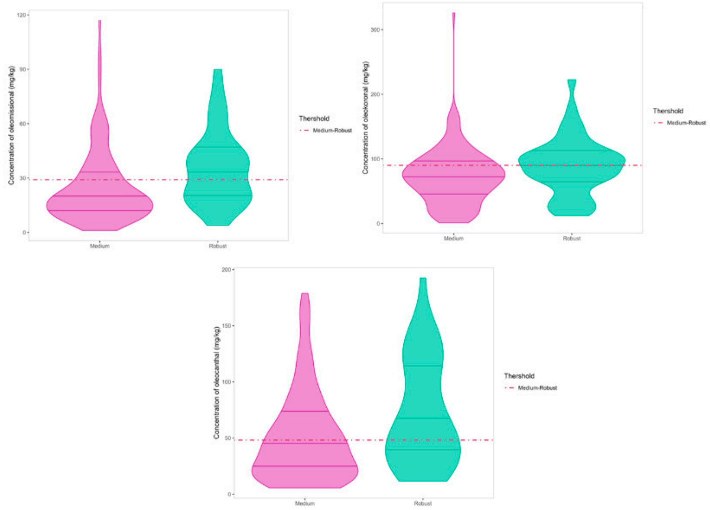

Figure S4. Violin plot for the concentration of secoiridoids (mg/kg) with significant differences between pungency groups. Threshold concentrations are included for discrimination of "*Medium*" and "*Robust*" (pink line).
